# Colorimetric Evaluation of a Reintegration via Spectral Imaging—Case Study: Nasrid Tiling Panel from the Alhambra of Granada (Spain)

**DOI:** 10.3390/s24123872

**Published:** 2024-06-14

**Authors:** Miguel Ángel Martínez-Domingo, Ana Belén López-Baldomero, Maria Tejada-Casado, Manuel Melgosa, Francisco José Collado-Montero

**Affiliations:** 1 Departamento de Óptica, Universidad de Granada, 18071 Granada, Spain; 2 Grupo de Investigación FQM381 de la Junta de Andalucía, Universidad de Granada, 18071 Granada, Spain; 3 Departamento de Pintura, Universidad de Granada, 18071 Granada, Spain

**Keywords:** color reintegration, hyperspectral imaging, color difference, visual appearance

## Abstract

Color reintegration is a restoration treatment that involves applying paint or colored plaster to an object of cultural heritage to facilitate its perception and understanding. This study examines the impact of lighting on the visual appearance of one such restored piece: a tiled skirting panel from the Nasrid period (1238–1492), permanently on display at the Museum of the Alhambra (Spain). Spectral images in the range of 380–1080 nm were obtained using a hyperspectral image scanner. CIELAB and CIEDE2000 color coordinates at each pixel were computed assuming the CIE 1931 standard colorimetric observer and considering ten relevant illuminants proposed by the International Commission on Illumination (CIE): D65 plus nine white LEDs. Four main hues (blue, green, yellow, and black) can be distinguished in the original and reintegrated areas. For each hue, mean color difference from the mean (MCDM), CIEDE2000 average distances, volumes, and overlapping volumes were computed in the CIELAB space by comparing the original and the reintegrated zones. The study reveals noticeable average color differences between the original and reintegrated areas within tiles: 6.0 and 4.7 CIEDE2000 units for the yellow and blue tiles (with MCDM values of 3.7 and 4.5 and 5.8 and 7.2, respectively), and 16.6 and 17.8 CIEDE2000 units for the black and green tiles (with MCDM values of 13.2 and 12.2 and 10.9 and 11.3, respectively). The overlapping volume of CIELAB clouds of points corresponding to the original and reintegrated areas ranges from 35% to 50%, indicating that these areas would be perceived as different by observers with normal color vision for all four tiles. However, average color differences between the original and reintegrated areas changed with the tested illuminants by less than 2.6 CIEDE2000 units. Our current methodology provides useful quantitative results for evaluation of the color appearance of a reintegrated area under different light sources, helping curators and museum professionals to choose optimal lighting.

## 1. Introduction

In the field of cultural heritage restoration, color reintegration is a specific treatment designed to improve the visual interpretation and appreciation of cultural artifacts. This treatment involves the application of paint or colored plaster to address material losses, contributing to a more cohesive and meaningful presentation of the object while respecting its significance [1]. Successful reintegration implies the use of colors that closely match the surrounding areas of the lacuna to accurately reconstruct the damaged paint surface. The reintegration must be recognizable to experts without attracting the viewer’s attention. However, there are some restored artworks in which misaligned retouches due to discolorations, darkening or color changes disrupt the visual perception of the restored artwork.

In the past, color studies of cultural heritage objects were mainly qualitative, lacking numerical specifications which may be very useful for curators and restorers. There is currently a growing trend toward quantitative assessments, as evidenced by recent studies [2,3] in which spectrophotometers are used. Spectral imaging is being used increasingly, as it offers spectral and spatial information at the same time. This leads to a more comprehensive understanding of the entire surface of artifacts, which has proven useful in evaluating different materials [4,5] and techniques [6] used in the restoration processes.

In addition, spectral information can be used to understand how the color appearance of cultural objects is modified under different illuminants. Illumination for museums and cultural heritage pieces has been previously studied, with the conclusion that there is no standardization in the practices of illumination, even though it is a factor of major importance for such institutions [7]. Furthermore, many researchers agree on the importance of using illumination systems such as LEDs, which are efficient and do not harm the valuable pieces exhibited in museums [8].

The illumination preferences of observers or museum visitors have also been widely studied through psychophysical experiments [9]. The optimal light sources to be used for observing a reintegrated piece are of great importance, as, depending on the sources selected, the original and reintegrated areas will appear more similar or more different.

The current work falls within the application of spectral images for qualitative and quantitative colorimetric analyses of cultural assets and the control and monitoring of conservation and restoration treatments. In this case, our research was aimed at evaluating color reintegrations using the technique of *rigatino* for a tiled skirting panel from the Nasrid period (1238–1492) displayed in the Museum of the Alhambra (Granada). This was a follow-up to previous research where a spectral color-imaging procedure was used for the colorimetric study of another artwork located in the cited Museum of the Alhambra, i.e., a portion of a Moorish epigraphic plasterwork frieze and a tiled skirting panel from the Nasrid Kingdom of Granada with later Moorish additions, under different illuminants [10]. The results demonstrated this approach to be an effective tool for heritage professionals when deciding which illumination to use in museums, or which conservation or restoration techniques best maintain the color appearance of the original piece under any illuminant.

In the current paper we will examine the impact of lighting on the visual appearance of a specific restored piece using hyperspectral imaging. Reflectance data was used to compute CIELAB color coordinates, considering ten relevant illuminants proposed by the International Commission on Illumination (CIE): D65 (the main CIE standard illuminant) plus nine white LEDs representative of current market-available LED sources [11]. The original and reintegrated areas of the tiles exhibited four main hues: blue, green, yellow, and black. For each hue, we computed the average distances, volumes, and overlapping volumes in the CIELAB space, comparing the original and the reintegrated zones. In addition, CIEDE2000 color differences with their lightness, chroma, and hue components were also computed. The Mean Color Difference from the Mean (MCDM) [12] was also determined for the restored and original areas in the four tiles to assess the heterogeneity of the CIELAB clouds of points corresponding to such areas.

## 2. Materials and Methods

### 2.1. The Cultural Heritage Piece

The artifact studied consists of a tiled skirting panel from the Nasrid period (Nasrid Kingdom of Granada, 1238–1492) on permanent display in the Museum of the Alhambra (Palace of Carlos V, Alhambra of Granada), with the inventory number R6267. It is a fragment of a skirting from the *rawda* of the Alhambra. The *rawda* was the royal cemetery or funerary pantheon located between the Nasrid Palaces and the Palace of Carlos V. The name *rawda* literally means “garden” but was often used to describe an enclosed burial ground with its associated architecture, surrounded by landscaped areas. Its foundation date is ambiguous, being attributed to Ismail I (beginning of the 14th century) or to Muhammad V. Mariano Contreras rediscovered the royal pantheon in 1887, but it was Leopoldo Torres Balbas who excavated and restored it in 1925. Between 1999 and 2000, it was excavated again, this time to support the *rawda* restoration work. Some of the tombstones from the royal tombs, panels of tiling (such as the one analyzed here) from the surrounding buildings, and other archaeological objects from the *rawda* are currently preserved in the Museum of the Alhambra [13,14].

**Figure 1:**
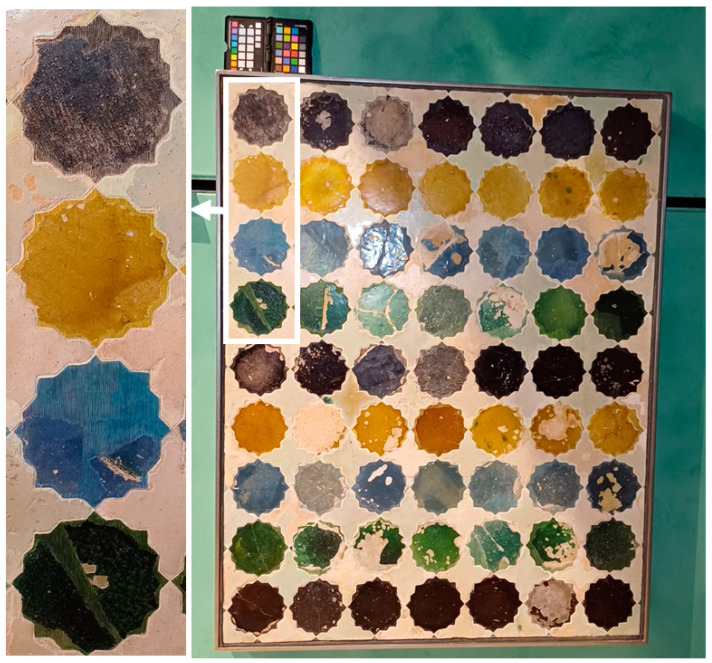
(**Right**) The complete art piece. (**Left**) Magnification of the studied area of the piece.

The tiled panel under study is a polychrome skirting made with a technique called *zellige*, in which a mosaic of glazed ceramic pieces, similar to tesserae, is created. In this case, its decorative geometric design is formed by sixteen-pointed stars, in different ranges of black, orange/yellow, blue, and green, linked by white crosses (see Figure 1). For this study, only the four (upper or bottom) half-stars (Figure 1, left column) that had been reintegrated were selected, one per hue.

As indicated above, the present paper focuses on color reintegrations of this panel, performed in glazed ceramic lacunae using the *Roman rigatino* technique, by means of a vertical line drawing in watercolor on a gypsum *stucco*. The EN 15898:2019 (3.5.7) [15] defines reintegration as an “action of restoration which consists of adding material for the reconstruction of appearance” of a cultural property. It is, therefore, a specific restoration treatment, meaning those “actions applied to a stable or stabilized object aimed at facilitating its appreciation, understanding, and/or use, while respecting and/or revealing its significance and the materials and techniques used” [13].

Therefore, the aim of reintegration is to complete or fill in a lost part (*lacuna*) of a heritage asset, both in terms of support and polychromy, or to integrate it aesthetically and complete it [16]. The *lacuna* or loss, in relation to an artwork or a heritage object, consists of “an interruption of the figurative fabric”, as C. Brandi has described it [17]. However, according to that same author, even worse than the loss is the negative effect that the lacuna has on the original work, because it has a form and a color which are foreign to the image depicted and, therefore, are not integrated into it. This is why, in many cases, it is necessary to remove the *lacuna* by means of a reintegration treatment in order to facilitate a correct and harmonious reading of the work.

Several international documents on conservation and restoration state that reintegration of lacunae or losses is a treatment that must follow specific criteria. In this respect, the *Carta di restauro* 1972 (Art. 7.1), written by C. Brandi [17], indicates that reintegration of historically verified small parts is admissible in the restoration of works using a different material from the original, but one which is harmonious and clearly distinguishable to the naked eye. The *Carta di restauro* 1987 [18] also indicates that reintegration works must be made with different, but compatible, materials from the original and chromatically integrated with the context [19].

Reintegration of lacunae can refer to support reintegration (structural reintegration) or color reintegration (retouching or in-painting). It is the latter that concerns us in this case study and consists of restoring the color of the lost pictorial layer or chromatic coating (in this case, a glazed coating). Different techniques are used for color reintegration, such as spot ink, dotting (in Italian, *puntinato*) or line drawing (in Italian, *rigatino* or *tratteggio*). All of these are methods that restore color losses in different ways: by applying a uniform pictorial layer (spot ink), by juxtaposed or overlayed color dots (dotting or *puntinato*), or by fine juxtaposed or overlayed color lines (line drawing, *rigatino* or *tratteggio*). In any case, the restored color must be in harmony with the original color, and it must also be distinguishable.

Reintegration using line drawing is based on “chromatic abstraction” and “chromatic selection”, in accordance with the names proposed by Baldini and Casazza [20,21,22]. Chromatic abstraction is a reintegration technique used when the image cannot be reconstructed due to significant losses, with the aim of obtaining a neutral ink based on the sum of the colors surrounding the loss; it is carried out by means of a grid of fine crossed lines of pure colors overlayed on top of each other. Chromatic selection, on the other hand, is a reintegration technique used when it is possible to restore both the shape and the lost color and is based on the use of fine lines of selected pure colors partially overlayed, producing a similar effect to the original color in the eye of the observer but without imitating it. In chromatic selection, the color lines are intended to reproduce the shape of the original brushstrokes in different directions, while the similar *Roman rigatino* follows an arrangement of vertical color lines. The latter is the method used in the color reintegrations analyzed in the present work.

### 2.2. Illuminants

**Figure 2:**
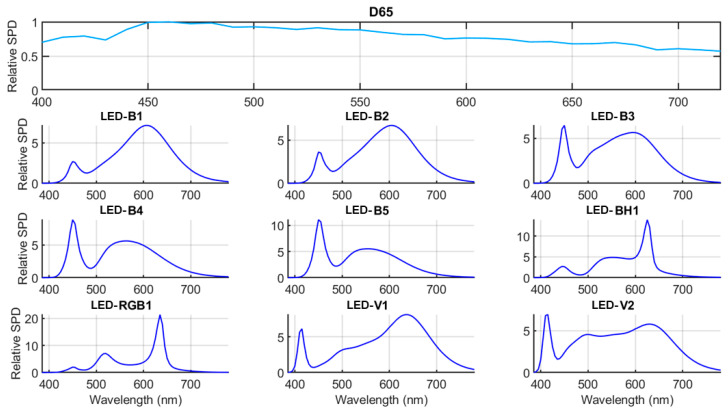
Spectral power distributions of the nine CIE LED illuminants and the standard CIE D65 illuminant.

The nine LED illuminants recently proposed by CIE [11] depict the spectral profiles of white LED lamps based on the measurements of several commercial LEDs. These profiles were derived from empirical assessments of spectral power distributions obtained from 1298 commercially available white LED sources [23,24]. Figure 2 shows the spectral power distribution of the nine CIE LED illuminants together with the CIE standard D65 illuminant included in this study.

Table 15 in [11] presents the key colorimetric characteristics of the nine CIE LED illuminants, including CIE x,y chromaticity coordinates, correlated color temperature (CCT), distance to Planckian locus (∆_uv_), CIE general color rendering index (R_a_), and the CIE 2017 color fidelity index (R_f_). Specifically, LED-B1 to LED-B5 characterize phosphor-converted blue LEDs at various CCTs, representing several widely used configurations. The LED-BH1 typifies white LEDs resulting from a blend of phosphor-converted blue and red LEDs, commonly referred to as blue-hybrid LEDs. The LED-RGB1 mirrors typical spectral compositions resulting from the mixture of red, green, and blue LEDs. Lastly, LED-V1 and LED-V2 portray spectral configurations typical of phosphor-converted violet LEDs at two distinct CCTs. It is noteworthy that white LED sources resembling LED-RGB1, LED-V1, and LED-V2 are not currently prevalent in usage [23]. Along with these nine CIE LED illuminants, CIE D65 has also been included in our study for the sake of comparison with the most widely used CIE standard illuminant.

### 2.3. Spectral Imaging System, Capture, and Processing

Hyperspectral images were achieved using a Resonon Pika L model (Resonon Inc., Bozeman, MT, USA) [25], located 35 cm from the sample, with a linear sensor of 900 pixels. The operational wavelength range spanned from 380 to 1080 nm. After the application of hardware binning, the spectral resolution obtained was 4.1 nm, while the spatial resolution was 0.12 mm/pixel. Given the emphasis of current research on colorimetric analyses, we considered the interval from 380 to 780 nm (visible range) exclusively.

**Figure 3:**
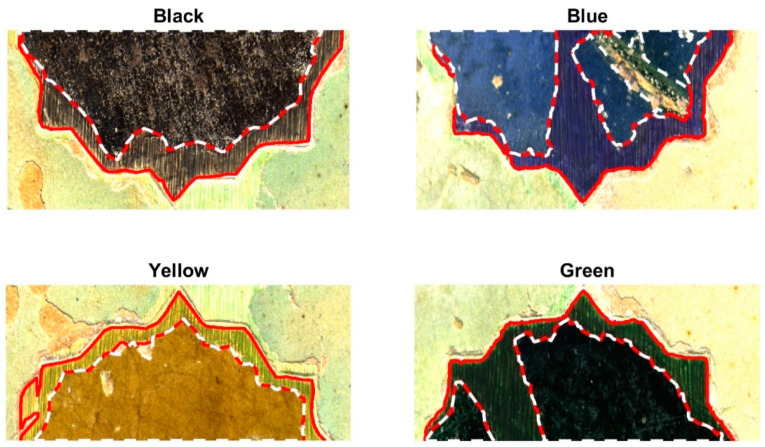
False RGB renderings of the black, blue, yellow, and green captured samples. The original and reintegrated areas are highlighted in dashed white and continuous red lines, respectively.

The sample was illuminated using two 36 Watt Cromalite Nanguan CN-600CSA LED panels (Nanguang Photo&Video Systems Co., Ltd., Shantou City, China) [26] positioned on still tripods at the left and right sides, along with a 500 Watt halogen lamp (Massive Mastec, Coral Gables, FL, USA) to increase illumination power in the NIR spectral region. Exposure time was fixed at 28 milliseconds per line, and ISO gain was set to 1. Two spectral images of the sample were captured, focusing on two different regions: one included the blue- and green-colored tiles, and the other the black- and yellow-colored tiles. The final widths of these images were 1780 and 1476 lines, and total capture times were approximately 50 s and 41 s, respectively, ensuring an adequate illuminance on the captured areas (700 lux on average at the center of the samples). False RGB images rendered from the spectral captures are shown in Figure 3. On the right part of the blue tile, an area can be observed that does not correspond to either the original or the reintegrated areas; it was therefore excluded from the calculations.

To address sensor spectral responses and the spatially and spectrally non-uniform illumination, as explained in [27] and utilized in [10,28], two additional images were captured under settings identical to those of the sample images ($I_{sample}$). These extra images, referred to as black ($I_{black}$) and gray ($I_{gray}$) images, allowed the correction process (termed “flat-field correction”), using the following Equation (1)

(1) $$I_{ref}\left(x,y,\lambda \right)=\frac{I_{sample}\left(x,y,\lambda \right)-I_{black}\left(x,y,\lambda \right)}{I_{gray}\left(x,y,\lambda \right)-I_{black}\left(x,y,\lambda \right)}\cdot ref_{gray}\left(\lambda \right)$$

where $I_{ref}$ is the flat-field-corrected reflectance image and $ref_{gray}$ is the known spectral reflectance of a gray homogeneous and nearly Lambertian surface used as reference [10].

### 2.4. Colorimetric Study

**Figure 4:**
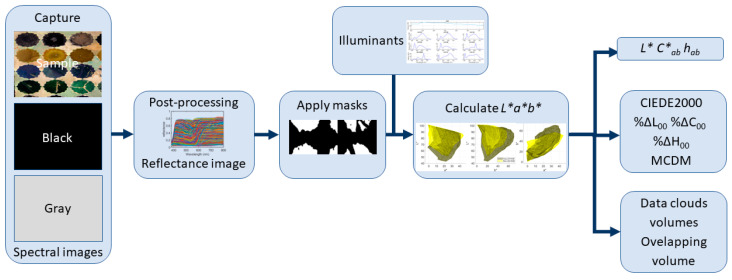
Workflow followed in this study.

Figure 4 shows the workflow followed once the spectral reflectance images have been retrieved.

#### 2.4.1. CIELAB Color Coordinates

After the spectral reflectance images were obtained, the CIELAB color coordinates were calculated pixelwise under the ten studied illuminants, assuming the CIE 1931 standard colorimetric observer. Spectral information only in the visible range, from 380 to 780 nm, was used to perform the calculation, and the spectral data were interpolated in a 5 nm step. Hence, for the calculation of the color information, the spectral interval used was Δλ = 5 nm. In addition to this, different manually built binary masks are applied to retrieve the color information separately for the original and reintegrated areas. In this way, we can analyze both separately and compare the two. These binary masks are images for which the only possible pixel values are 1, meaning that the pixel belongs to a given class, and 0, meaning that the pixel does not belong to this class. Eight classes are built, representing the four hues studied—black, blue, green, and yellow—in the two conditions: original and reintegrated. Hence, 8 different binary masks were built.

In some cases, transforming L*a*b* values into L* C*_ab_ *h*_ab_ [11] values can help us to understand colors from the point of view of perceptual attributes that are usually referred to as lightness, chroma, and hue.

#### 2.4.2. CIEDE2000 Color Differences and Components

The current ISO/CIE recommendation for measuring color differences under reference conditions is the CIEDE2000 color-difference formula [29]. For the L*a*b* coordinates corresponding to the original and reintegrated areas of each hue, we computed their geometrical centers (mean colors), and then the CIEDE2000 color differences between these two mean colors. This simple computation gives us a first glance at how different or similar the two colors are on average, and over an extensive area. This is akin to what a point- or area-based measurement device such as a colorimeter or a spectrophotometer would yield as a result for homogeneous samples.

Total color differences in CIEDE2000 units can also be expressed by the corresponding variations in the three main perceptual attributes of color: lightness differences ($∆L_{00}$), chroma differences $(∆C_{00}$), and hue differences ($∆H_{00}$) [30]. These three types of color differences can be given as percentages. For example, the percentage of lightness difference is computed as $\%∆L_{00}=100{\left(∆L_{00}/∆E_{00}\right)}^{2}$, and similarly the percentages of chroma and hue differences. Consequently, based on this definition, it can be deduced that

(2) $$\%∆L_{00}+\%∆C_{00}+\%∆H_{00}=100\;\;\;$$

Once the L*a*b* coordinates of the pixels are computed for each class, we have clouds of L*a*b* points and can proceed to calculate different parameters for comparing the same colors in the two possible conditions, i.e., the original vs. the reintegrated areas. We can thereby assess the extent to which the reintegration procedure followed for this piece has resulted in a final appearance which is similar or different to its original appearance under the different illuminants studied. With this methodology, selecting optimal LED illumination would be possible for a given piece of art, depending on whether we want to highlight or disguise the reintegrated areas.

#### 2.4.3. CIELAB Data Clouds, Volumes, and Percentage of Overlapping Volume

The L*a*b* coordinates of each pixel in both the reintegrated and original areas can be represented as data clouds in the CIELAB space. This representation improves the comprehension of the 3D distribution of the pixel colors. In addition, the volume of each data cloud can be computed using Delaunay’s triangulation method [31] with infinite radius, together with an auxiliary algorithm based on the alpha-shape concept [32]. We can then calculate the percentage of overlapping volume between the two L*a*b* clouds (i.e., original and reintegrated areas) for each color tile. To do this, we consider 100% as the sum of both volumes, and compute the volume of the overlapping three-dimensional region between the two clouds. The percentage of overlap is determined by comparing the volume of the overlap against the sum of both volumes.

#### 2.4.4. Mean Color Difference from the Mean (MCDM)

Once the mean color differences and the volume of the L*a*b* clouds are studied, we can gain an insight into how extensive (and hence heterogeneous) the two samples are that correspond to the original and reintegrated areas of a given hue. However, these two samples could still have the same mean color and the same cloud volume (100% overlap), and have different inner distributions, which in turn could result in having still different appearances. In order to quantify this difference in inner distributions as well, the Mean Color Difference from the Mean (MCDM) is calculated as shown in Equation (3) [12], using the $∆E_{00}$:

(3) $$MCDM=\;\frac{\sum_{i=1,n}∆E_{00}\left(L_{i}^{*}a_{i}^{*}b_{i}^{*},\;\bar{L^{*}a^{*}b^{*}}\right)}{n}$$

**Figure 5:**
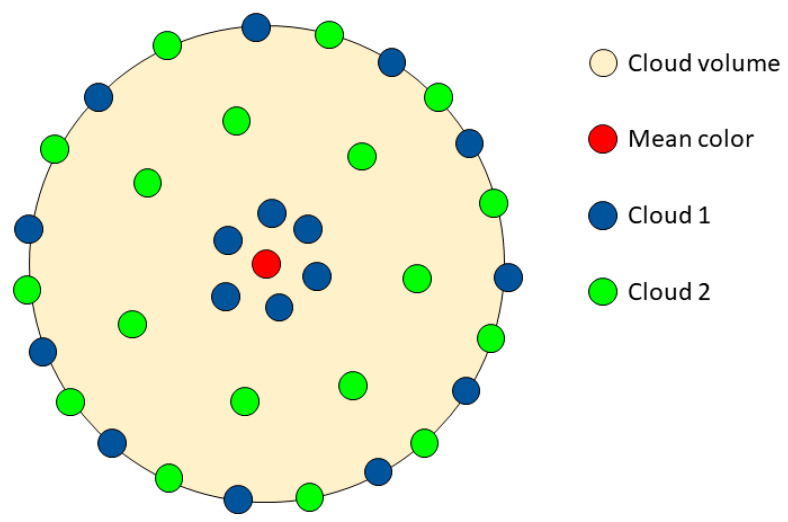
Scheme of two theoretical L*a*b* clouds with identical mean color and volume, but different MCDM values. Specifically, in this example the MCDM for cloud 1 is lower than the MCDM for cloud 2.

For the calculation of this parameter, we first compute the mean color. Then, we calculate the CIEDE2000 color difference of each point of the cloud in relation to the mean color. After this, we compute the mean value across all these color differences. A lower value of MCDM means that the inner distribution of points in the cloud is closer to the mean color (its center of gravity). However, larger values of the MCDM mean that the distribution of points is more spread out within the cloud volume. An example of the usefulness of the MCDM measurement can be seen in Figure 5.

## 3. Results and Discussion

### 3.1. Mean Reflectance Spectra, Color Coordinates, and Variability

**Figure 6:**
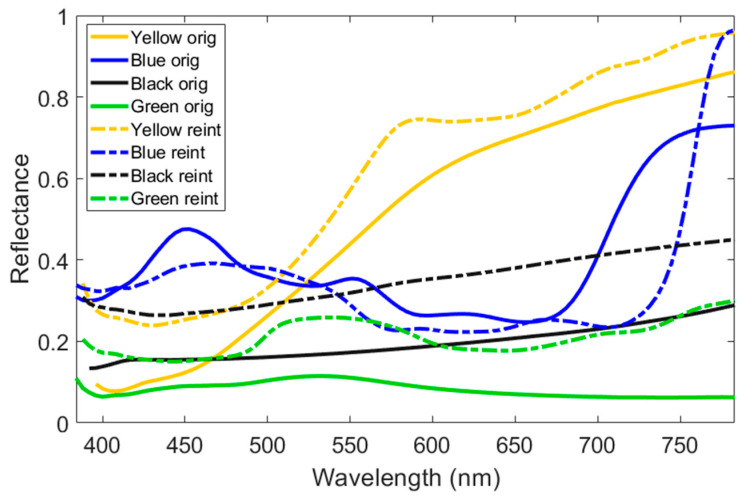
Average reflectance spectra of the original (continuous lines) and reintegrated (dashed lines) areas for the four tiles (yellow, blue, black, and green).

The average reflectance spectra for both the original (continuous lines) and reintegrated (dashed lines) areas of the four tiles are presented in Figure 6.

The yellow, blue, and green tiles exhibit a clear change in the shape of the reflectance spectra when comparing the reintegrated areas to the original ones. In contrast, the black tile spectrum seems to change only in amplitude, being higher in the reintegrated area. This may be due to one or both of the following factors: first, the restorer may have used pigments different from those in the original area; second, the reflectance of the pigments may have changed shape due to aging. Another possibility is that the restorer decided to use lighter colors for the reintegration of the two darker tiles (black and green), making the reintegrated areas visible at close range.

The mean and standard deviations (SDs) of L*, C*_ab_, and *h*_ab_ color coordinates for the four tiles (original and reintegrated areas), and the ten illuminants under consideration are presented in Table 1.

In Table 1, the hab values for the original and reintegrated areas can be grouped into four main hue angles, corresponding to a yellow (~60–80°), blue (~230–270°), black (with a high variability in hue angle due to low chromaticity), or green (~130–170°) hue. Generally, in comparing both the original and reintegrated areas of the tiles, we observe a higher value of L* in the reintegrated area. This might be explained by the way in which the reintegration process is conducted. The lacuna is filled with *stucco* and painted with a *rigatino* technique that uses drawn vertical lines. These lines reveal the beige *stucco* underneath. Therefore, while the green and black hues of the original tiles have L* values around 36 and 46, respectively, these are around 54 and 63 for the reintegrated areas. The effect is less noticeable in the yellow tile, as the original color is more similar to the color of the *stucco*.

In general, standard deviations (in parentheses in Table 1) show similar values when comparing the reintegrated with the original areas or different illuminants for the same area. In studying the color of the tiles, the L* and hab coordinates are very uniform, especially for the yellow and blue tiles. In particular, the black tile exhibits significant variations in both the L* and hab coordinates compared to the other three tiles. This might be due to the fact that the original area of the black tile is more deteriorated than the other original or reintegrated areas (see Figure 1). This difference in lightness and hue can also be seen in Figure 1.

The variation in color coordinates for all pixels in both the original and reintegrated areas can be examined not only through the standard deviations presented in Table 1 but through the Mean Color Difference from the Mean (MCDM) [12]. The MCDM in the CIEDE2000 units for the original and reintegrated areas under all tested illuminants is shown in Table 2. The mean and standard deviation (SD) across all illuminants are also provided in order to quantify the impact of the illuminant on the color of each tile.

From the values in Table 2, it is worth noting that for the yellow, blue, and green tiles, the original colors are more evenly perceived than the reintegrated ones, while the opposite is found for the black tile, possessing a reintegrated area which is more uniform than the original (as can be seen in Figure 1). In this sense, the least homogeneous tile color is also black, with MCDM values around 13 and 12 CIEDE2000 units for the original and reintegrated tiles, respectively, while the most homogeneously perceived is yellow, with MCDM values around 4 CIEDE2000 units for both the original and the reintegrated tiles. This could again be attributed to the deterioration of the black tile (see Figure 1). In contrast, changing the illuminant appears to have only a minimal effect on the perceived homogeneity of the tile’s color. Similar values of MCDM are consistently obtained for all tiles under the studied illuminants. This observation is supported by the standard deviations computed in the last column of Table 2, all of which are below 0.12 CIEDE2000 units.

### 3.2. CIEDE2000 Color Differences, Original vs. Reintegrated, and Components

**Figure 7:**
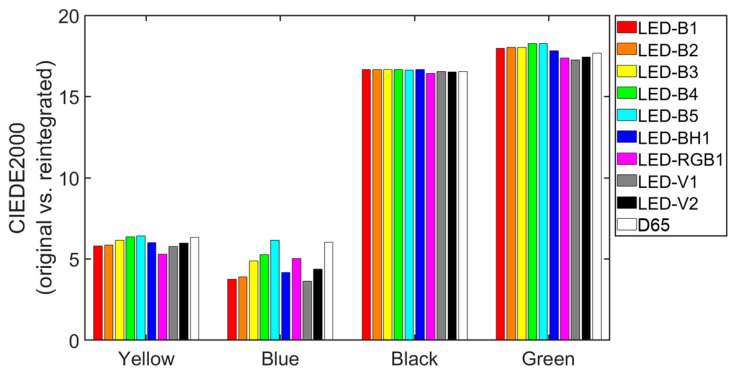
Average CIEDE2000 color differences (original vs. reintegrated areas) for the four tiles (yellow, blue, black, and green) under the ten CIE illuminants.

Figure 7 shows the mean CIEDE2000 color differences between the original and reintegrated areas under each illuminant.

When comparing the results for four color tiles in Figure 7, the CIEDE20000 values for the black and green tiles are about three times greater than those for the yellow and blue tiles. That is, the two darkest tiles (as seen in the L* coordinate of Table 1) show a greater difference between the original and reintegrated areas. It is worth noting that the underlying, beige-colored *stucco* contributes to the higher differences in L* coordinates between the two areas. Additionally, dark colors tend to be more achromatic, with lower values of C*_ab_, leading to significant variations in *h*_ab_ coordinates. Regarding the influence of the illuminants, the lowest CIEDE2000 value is achieved with the blue tile and the LED-V1 illuminant. For this tile, the color difference ranges from 3.6 CIEDE2000 units with the LED-V1 illuminant to 6.2 CIEDE2000 units with the LED-B5 illuminant. In contrast, the tile for which the influence of the illuminant is least noticeable is the black one, with very similar CIEDE2000 values for all the illuminants studied. The highest CIEDE2000 values obtained for the black and green tiles are found under the LED-B4 illuminant (16.7 and 18.3 CIEDE2000 units, respectively), while for the yellow and blue tiles these are found under the LED-B5 illuminant (6.4 and 6.2 CIEDE2000 units, respectively), as shown in Figure 7. Based on the results presented in Table 1 of [33], visual color thresholds for human observers with normal color vision fall within the range of 0.4–0.7 CIEDE2000 units, considering the threshold experiments (TCD) mentioned in [34]. According to this result, the original and reintegrated areas in the current work will consistently be perceived as different by observers with normal color vision under all tested illuminants.

In order to know the percentage of total color difference corresponding to the differences in the three main color attributes (see Section 2.4.2), Table 3 is presented.

The values presented in Table 3 show that for the yellow, black, and green tile colors and all illuminants, the total color difference is mainly attributable to an increase of lightness in the reintegrated areas. However, for the blue tile, the total color difference is mostly produced by differences in hue angle when this tile is illuminated by LED-B3, LED-B4, LED-B5, LED-RGB1, and D65 illuminants. In addition, for the blue tile, when LED-RGB1, LED-V1, and LED-V2 are used, chroma differences also play an important role in the total color difference. Regarding the chroma attribute, for the blue, black, and green tiles, we see generally less influence in total color difference than for the yellow tile. These results show that there is no single illuminant that consistently provides similar results for all tiles and, therefore, the color differences between the original and reintegrated areas will depend on the tile considered. From all indications, it is clear that for all tiles and illuminants the average color differences between the original and reintegrated areas are fully perceptible, given the color threshold value of 0.4–0.7 CIEDE2000 units [33,34].

Since the MCDM values (Table 2) have the same order of magnitude as average CIEDE2000 color differences (Figure 7), it is interesting to represent in CIELAB space the color coordinates of all pixels in both the original and reintegrated areas. This representation is necessary for understanding the 3D distribution and overlapping of the two data clouds (where data clouds are defined as the L*a*b* coordinates of each pixel of the original and reintegrated areas), as discussed in the Section 3.3.

### 3.3. CIELAB Data Clouds, Volumes, and Overlapping

After discussing the color obtained for each tile, assessing its spatial distribution in relation to the mean using the MCDM, and computing the average color difference between the original and reintegrated areas under all illuminants, the volume of the color gamut for each tile and area (original/reintegrated) under all tested illuminants is obtained (Table 4). The color gamut is obtained by representing the L*a*b* coordinates of each pixel in the CIELAB space.

Higher gamut volume indicates higher color heterogeneity. Of the four tile colors, the original area of the black tile has the biggest gamut, while the original area of the blue tile has the smallest (see Table 4). This shows that the original black tile is the least uniform in terms of color, as can be seen in Figure 1. Except for the blue tile, the volume obtained for the reintegrated area is consistently smaller than that of the original under all illuminants. This indicates that the reintegrated area is more uniform in color, possibly due to a deterioration in the original areas. For the black tile, the gamut volume of the original area is more than double that of the reintegrated area, making it the one that shows the greatest difference between the original and reintegrated areas. In contrast, the yellow tile presents the most similar gamut volumes when comparing the original and reintegrated areas. This may be because the original color of this tile is similar to that of the *stucco* that can be seen beneath it in the reintegrated area due to the *rigatino*. Therefore, to an average observer, the yellow tile will appear more color-consistent than the other tiles, as it is more difficult to distinguish between the original and reintegrated areas. If we compare different illuminants, we can see that LED-RGB1 and D65 provided the biggest gamuts, while LED-B1 and LED-B5 provided the smallest gamuts, depending on the color and area of the tile. To quantify the impact of the illuminant for each area and tile, the coefficient of variation (CV) is presented in the last column of Table 4. It shows a maximum value of 12.8 for the original area of the green tile, and a minimum value of 7.7 for the reintegrated area in the yellow tile.

**Figure 8:**
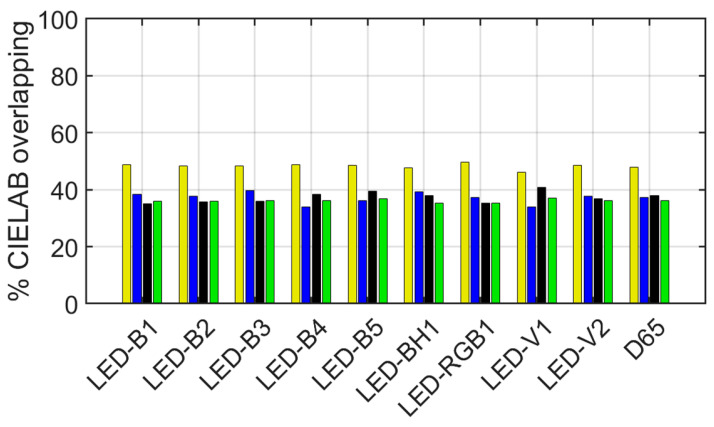
Percentage of L*a*b* overlapping volume between the original and reintegrated areas of the yellow, blue, black, and green tiles (distinguished by the colors of the bars) under the ten CIE illuminants.

Figure 8 shows the percentage of overlap of the CIELAB volumes or gamuts for the original and reintegrated areas of each tile across all the illuminants studied.

The overall overlap values obtained are not very high, between 34% and 50%, so that at a working distance it can be assumed that the original and reintegrated areas will be visually perceived as different, regardless of the tile and illuminant. The maximum value of overlapping volume (49.6%) is achieved for the yellow tile and the LED-RGB1 illuminant, while the minimum value (34.1%) is obtained for the blue tile with the LED-V1. Note in Figure 8 a similar trend for all tiles and illuminants, with the yellow tile providing the highest value of overlapping volume. In addition, the illuminant does not influence the overlapping volume greatly, with a maximum overlap difference of 5.7% between the LED-B3 and the LED-V1 for the blue tile.

**Figure 9:**
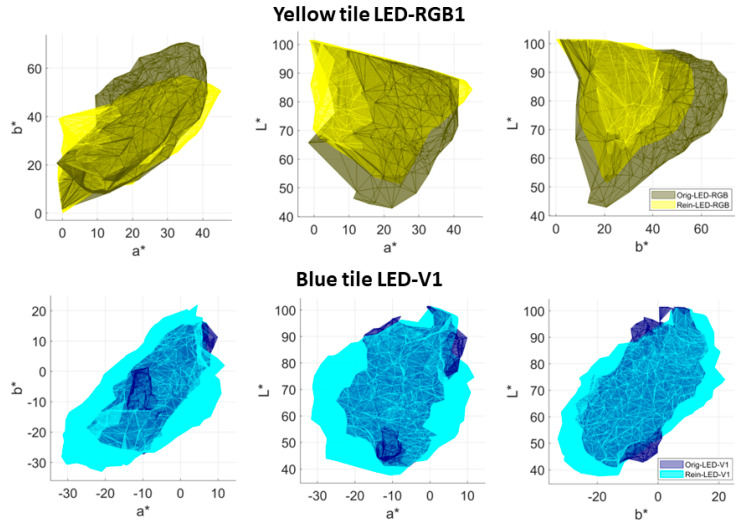
2D projections from the L*a*b* color space representing the color gamuts of the original (light) and reintegrated (dark) areas for the yellow (**upper row**) and blue (**lower row**) tiles, using the LED-RGB1 and LED-V1 illuminants, respectively.

Finally, to evaluate and compare the color gamuts in the CIELAB color space across different tiles and illuminants, these are represented as solids using the alpha shape algorithm [32]. As an illustrative example, we selected the largest and smallest overlapping volumes and show their 2D projections in the a*b*, a*L*, and b*L* planes in Figure 9. This Figure provides a comparison between the color gamuts of the original (dark) and reintegrated (light) areas of the yellow (upper row) and blue (lower row) tiles for the LED-RGB1 and LED-V1 illuminants, respectively.

The color gamuts of the original and reintegrated areas are more similar in the yellow tile (Figure 9 first row) than in the blue tile (Figure 9 second row). In the yellow tile, we can mainly see that the gamut of the original area is shifted towards higher values of the b* coordinate compared to the gamut of the reintegrated area, which implies a higher value of chroma for the original tile. In the blue tile, the gamut of the reintegrated area is wider than that of the original area. This indicates that for the blue tile, the original area is more homogeneous in terms of color than the reintegrated area.

## 4. Conclusions

In this work, a tiled skirting panel from the Nasrid period (the Nasrid kingdom of Granada, 1238–1492), permanently on display in the Museum of the Alhambra (Spain), has been studied in order to quantify the color differences between the original and reintegrated areas of the piece under different illuminants. For this purpose, spectral images from 380 to 1080 nm were captured, and the CIELAB color coordinates of each area were calculated. Ten illuminants (the CIE standard D65, plus nine white LEDs proposed by the CIE) and four tiles with different hues (blue, green, yellow, and black) were examined. 

Assuming the CIE 1931 standard colorimetric observer for each hue and illuminant, we computed the average L*, C*_ab_, and *h*_ab_ color coordinates, along with the average CIEDE2000 color differences between the original and reintegrated areas, with their corresponding lightness, chroma, and hue components. Additionally, we analyzed the MCDM (Mean Color Difference from the Mean), gamut volumes, and overlapping volumes in CIELAB by comparing the original and reintegrated areas.

From the CIEDE2000 values, the color differences between the original and reintegrated areas are shown to vary by a factor of three in the samples studied; specifically, in the yellow and blue tiles, this is around 5 CIEDE2000 units, while in the black and green tiles, it is around 15 CIEDE2000 units. In any case, these color differences are clearly noticeable, being above the color discrimination threshold of the human eye. The percentage of overlapping volumes of the original and reintegrated areas is low, ranging between 35% and 50%. Thus, based on the CIEDE2000 values and the overlapping volumes, we can conclude that the original and reintegrated areas would be perceived as visually different in all four tiles. In fact, this is the main goal of restorers in near vision, which agrees with our current instrumental measurements.

Our colorimetric results also corroborate that the original area of the black tile exhibits more signs of deterioration than the original or reintegrated areas of the other tiles. The similarity of the reintegrated area to the original tiled area is only marginally influenced by the chosen illuminant. Therefore, there would be no optimal illuminant for minimizing (or maximizing) the discrimination of the original and reintegrated areas, and so the optimal illuminant can be freely chosen, for example, according to aesthetic criteria.

The techniques and results discussed in this paper offer curators and museum professionals some tools to make informed decisions on the most suitable illumination for displaying reintegrated pieces in museums. Our current methodology provides quantitative results that could be valuable for objectively evaluating the color appearance of a reintegrated area under various light sources. In addition, the use of hyperspectral imaging has enabled the colorimetric evaluation of the piece as a whole (e.g., with regard to color gamuts), obtaining information not only for specific points, but for the entire piece. This methodology is not limited to the case study shown; it can be applied to different cultural heritage pieces, including various materials, colors, and types of artifacts such as tiles, plasterworks, paintings, and ceramics. By employing this methodology, museum professionals can select the type of illumination based on the desired effect and appearance of both original and restored areas of their heritage pieces and artworks.

## Figures and Tables

**Table 1:** Mean and standard deviations (in parentheses) of L* C*_ab_ *h*_ab_ color coordinates for original (Orig) and reintegrated (Reint) areas of the tiles with yellow, blue, black, and green hues under the ten tested CIE illuminants.

		Yellow		Blue		Black		Green	
		Orig	Reint	Orig	Reint	Orig	Reint	Orig	Reint
LED-B1	L*	77.7 (6.8)	85.1 (6.8)	62.4 (7.3)	58.5 (7.0)	46.5 (16.1)	63.9 (12.2)	36.5 (8.3)	53.9 (10.2)
	C*_ab_	56.1 (7.9)	46.2 (7.8)	19.4 (4.4)	20.4 (6.8)	7.2 (5.4)	9.7 (5.9)	8.7 (3.5)	15.6 (6.2)
	hab	75.1 (4.0)	76.1 (4.9)	248.0 (15.6)	241.5 (29.0)	114.3 (109.5)	79.4 (63.0)	174.2 (26.8)	133.8 (18.0)
LED-B2	L*	77.2 (6.8)	84.7 (6.8)	62.6 (7.2)	58.8 (7.0)	46.4 (16.0)	63.7 (12.1)	36.7 (8.3)	54.0 (10.2)
	C*_ab_	55.8 (7.9)	45.8 (7.7)	19.2 (4.4)	20.1 (6.5)	7.1 (5.3)	9.5 (5.8)	8.9 (3.7)	16.2 (6.4)
	hab	75.3 (4.1)	76.1 (5.1)	249.1 (15.9)	241.1 (29.3)	115.6 (110.3)	79.9 (63.6)	172.8 (26.7)	133.7 (17.4)
LED-B3	L*	75.9 (6.7)	83.4 (6.7)	63.2 (7.1)	59.6 (7.0)	46.2 (15.9)	63.4 (12.0)	37.1 (8.3)	54.4 (10.2)
	C*_ab_	54.9 (8.0)	44.2 (7.4)	17.9 (4.3)	18.7 (5.7)	7.0 (5.2)	9.1 (5.5)	9.6 (4.4)	17.8 (6.9)
	hab	77.9 (4.4)	77.3 (5.7)	254.6 (17.6)	240.5 (31.1)	127.8 (116.1)	86.2 (70.2)	168.7 (26.7)	134.4 (15.7)
LED-B4	L*	75.3 (6.7)	83.0 (6.7)	63.5 (7.1)	59.8 (7.0)	46.1 (15.9)	63.3 (11.9)	37.3 (8.2)	54.7 (10.2)
	C*_ab_	54.2 (8.0)	43.2 (7.1)	17.5 (4.4)	17.6 (5.1)	6.7 (5.0)	8.7 (5.3)	9.6 (4.8)	19.0 (7.5)
	hab	83.0 (4.6)	82.4 (5.8)	262.5 (18.4)	246.7 (32.6)	133.6 (115.3)	92.2 (69.4)	166.4 (27.5)	134.3 (14.0)
LED-B5	L*	74.5 (6.7)	82.2 (6.7)	63.8 (7.1)	60.3 (7.0)	46.0 (15.9)	63.1 (11.9)	37.4 (8.2)	54.7 (10.2)
	C*_ab_	52.4 (7.8)	41.7 (6.9)	17.0 (4.4)	16.8 (4.7)	6.4 (4.8)	8.3 (5.1)	9.7 (5.0)	19.7 (7.8)
	hab	86.2 (4.8)	85.1 (6.1)	269.0 (19.3)	249.4 (33.7)	137.9 (115.4)	96.3 (69.5)	166.1 (27.6)	135.7 (13.1)
LED-BH1	L*	77.2 (6.8)	84.3 (6.8)	62.7 (7.2)	59.0 (7.0)	46.5 (16.0)	63.7 (12.1)	36.7 (8.3)	54.0 (10.2)
	C*_ab_	58.1 (8.1)	46.1 (7.8)	18.9 (4.4)	20.2 (6.5)	7.7 (5.6)	10.0 (6.1)	10.3 (4.2)	17.4 (6.6)
	hab	73.7 (4.2)	74.1 (5.7)	245.3 (16.8)	235.5 (30.3)	124.4 (116.8)	82.7 (73.0)	170.7 (25.1)	137.0 (17.1)
LED-RGB1	L*	76.3 (6.7)	82.7 (6.8)	62.8 (7.3)	60.2 (6.9)	46.5 (15.9)	63.6 (12.0)	36.7 (8.3)	53.8 (10.0)
	C*_ab_	54.5 (7.3)	44.8 (8.3)	19.2 (4.2)	22.9 (8.6)	8.4 (5.7)	10.6 (6.0)	12.6 (4.6)	17.4 (5.7)
	hab	60.3 (3.8)	59.9 (6.2)	234.1 (16.2)	221.5 (27.7)	98.7 (113.3)	63.2 (66.2)	177.3 (20.3)	146.5 (19.3)
LED-V1	L*	77.3 (6.8)	84.4 (6.8)	62.5 (7.2)	59.0 (6.9)	46.5 (16.0)	63.8 (12.1)	36.5 (8.3)	53.7 (10.1)
	C*_ab_	56.1 (7.3)	45.4 (7.8)	16.2 (4.0)	19.9 (6.7)	7.8 (5.3)	10.0 (6.1)	10.2 (3.9)	15.3 (5.6)
	hab	71.1 (4.0)	71.4 (5.4)	235.7 (16.7)	232.2 (30.3)	125.8 (119.8)	88.4 (88.8)	168.2 (22.9)	139.1 (19.3)
LED-V2	L*	75.5 (6.7)	82.8 (6.8)	63.3 (7.1)	60.0 (6.9)	46.2 (15.9)	63.4 (11.9)	37.0 (8.3)	54.2 (10.1)
	C*_ab_	53.4 (7.3)	43.4 (7.4)	15.7 (3.6)	19.1 (6.2)	7.3 (4.9)	9.3 (5.6)	10.6 (4.4)	16.9 (6.2)
	hab	71.6 (4.3)	70.7 (6.1)	241.2 (17.4)	230.9 (30.6)	128.0 (122.1)	86.6 (86.5)	166.2 (21.9)	137.3 (16.8)
D65	L*	74.1 (6.7)	81.5 (6.7)	63.9 (7.0)	60.8 (6.9)	46.0 (15.8)	63.1 (11.8)	37.3 (8.2)	54.5 (10.1)
	C*_ab_	51.0 (7.3)	40.7 (6.8)	14.8 (3.5)	16.9 (4.8)	6.9 (4.6)	8.5 (5.0)	10.8 (5.0)	18.8 (7.1)
	hab	78.9 (4.8)	77.0 (6.9)	257.0 (19.8)	237.1 (33.4)	138.2 (123.5)	92.7 (83.4)	165.1 (23.1)	137.7 (14.0)

**Table 2:** Mean Color Differences from the Mean (MCDM) in CIEDE2000 units for original (Orig) and reintegrated (Reint) areas of the four tiles under the ten tested CIE illuminants. Mean ± standard deviation (SD) across all illuminants for each area and tile color are shown in the last column.

		LED- B1	LED- B2	LED- B3	LED- B4	LED- B5	LED- BH1	LED- RGB1	LED- V1	LED- V2	D65	Mean ± SD
Yellow	Orig	3.6	3.6	3.7	3.8	3.8	3.7	3.6	3.6	3.7	3.9	3.71 ± 0.10
	Reint	4.5	4.5	4.5	4.5	4.5	4.6	4.6	4.6	4.6	4.6	4.56 ± 0.04
Blue	Orig	5.8	5.8	5.8	5.9	5.9	5.9	5.9	5.8	5.7	5.8	5.84 ± 0.04
	Reint	7.0	7.1	7.2	7.2	7.3	7.2	7.3	7.2	7.3	7.4	7.23 ± 0.11
Black	Orig	13.2	13.2	13.2	13.1	13.1	13.4	13.4	13.4	13.2	13.1	13.23 ± 0.12
	Reint	12.2	12.1	12.2	12.1	12.1	12.2	12.2	12.3	12.2	12.2	12.18 ± 0.05
Green	Orig	10.8	10.8	10.9	11.0	11.0	10.9	10.9	10.8	10.8	10.9	10.89 ± 0.07
	Reint	11.3	11.3	11.3	11.3	11.3	11.4	11.4	11.3	11.3	11.3	11.33 ± 0.03

**Table 3:** Percentages of lightness, chroma, and hue differences in the total CIEDE2000 color difference (original—reintegrated) for tiles with four hues under the ten tested CIE illuminants. Values with (*) indicate that the corresponding attribute of the reintegrated tile increases with respect to that of the original.

		Yellow	Blue	Black	Green
LED-B1	%Δ*L*_00_	72.0 *	85.6	96.3 *	83.8 *
	%Δ*C*_00_	27.5	0.4 *	2.5 *	5.1 *
	%Δ*H*_00_	0.5 *	14.0	1.2 *	11.1
LED-B2	%Δ*L*_00_	71.9 *	77.2	96.3 *	83.7 *
	%Δ*C*_00_	28.0	0.2 *	2.5 *	5.6 *
	%Δ*H*_00_	0.1 *	22.6	1.2 *	10.7
LED-B3	%Δ*L*_00_	68.7 *	42.8	96.2 *	84.2 *
	%Δ*C*_00_	30.3	0.0	2.2 *	7.5 *
	%Δ*H*_00_	1.0	57.2	1.6 *	8.3
LED-B4	%Δ*L*_00_	68.4 *	36.1	96.0 *	83.3 *
	%Δ*C*_00_	30.7	2.6	2.3 *	10.0 *
	%Δ*H*_00_	0.9	61.3	1.7 *	6.7
LED-B5	%Δ*L*_00_	68.2 *	22.5	96.0 *	82.9 *
	%Δ*C*_00_	30.0	4.9	2.2 *	11.5 *
	%Δ*H*_00_	1.8	72.6	1.8 *	5.6
LED-BH1	%Δ*L*_00_	62.4*	62.2	96.0 *	85.5 *
	%Δ*C*_00_	37.6	1.3 *	2.5 *	5.2 *
	%Δ*H*_00_	0.0	36.6	1.5 *	9.3
LED-RGB1	%Δ*L*_00_	64.8 *	19.5	97.1 *	88.3 *
	%Δ*C*_00_	33.7	20.3 *	1.9 *	1.9 *
	%Δ*H*_00_	1.5	60.2	1.0 *	9.8
LED-V1	%Δ*L*_00_	66.4 *	70.5	96.9 *	90.0 *
	%Δ*C*_00_	33.6	24.6 *	1.9 *	2.5 *
	%Δ*H*_00_	0.0	4.9	1.2 *	7.5
LED-V2	%Δ*L*_00_	68.4 *	41.2	96.9 *	88.5 *
	%Δ*C*_00_	29.4	21.4 *	1.7 *	4.0 *
	%Δ*H*_00_	2.2	37.4	1.4 *	7.5
D65	%Δ*L*_00_	65.6 *	20.3	96.5 *	86.7 *
	%Δ*C*_00_	29.5	3.1 *	1.5 *	7.2 *
	%Δ*H*_00_	4.9	76.6	2.0 *	6.1

**Table 4:** Gamut volumes (CIELAB cubic units) for the four tiles (original and reintegrated areas) under the ten tested illuminants. The last column shows the coefficient of variation (CV) across all illuminants tested for each area and tile.

		LED- B1	LED- B2	LED- B3	LED- B4	LED- B5	LED- BH1	LED- RGB1	LED- V1	LED- V2	D65	CV (%)
Yellow	Orig	27,005	28,075	31,740	31,563	31,477	34,349	35,823	30,350	35,322	36,011	9.9
	Reint	23,651	24,527	26,629	24,686	24,220	28,555	29,163	28,056	28,000	27,627	7.7
Blue	Orig	14,586	15,179	15,972	14,154	13,675	18,813	19,690	14,656	16,038	15,347	12.5
	Reint	28,163	28,832	30,932	30,286	29,475	34,485	36,581	33,187	31,672	31,531	8.3
Black	Orig	41,040	42,807	49,749	46,390	44,568	52,967	57,036	44,472	47,799	49,455	10.2
	Reint	17,778	18,446	23,660	22,556	21,948	24,442	22,671	21,087	22,323	24,918	10.7
Green	Orig	35,686	36,232	39,312	36,389	34,292	48,304	48,887	43,367	41,524	39,590	12.8
	Reint	26,685	27,201	28,262	26,404	24,926	33,241	35,494	30,065	30,297	27,916	11.3

## Data Availability

The data that support the findings of this study are available upon request.
